# *TP53* Mutation Spectrum in Smokers and Never Smoking Lung Cancer Patients

**DOI:** 10.3389/fgene.2016.00085

**Published:** 2016-05-11

**Authors:** Ann R. Halvorsen, Laxmi Silwal-Pandit, Leonardo A. Meza-Zepeda, Daniel Vodak, Phuong Vu, Camilla Sagerup, Eivind Hovig, Ola Myklebost, Anne-Lise Børresen-Dale, Odd T. Brustugun, Åslaug Helland

**Affiliations:** ^1^Department of Cancer Genetics, Institute for Cancer Research, Oslo University Hospital – The Norwegian Radium HospitalOslo, Norway; ^2^Department of Tumour Biology, Institute for Cancer Research, Oslo University HospitalOslo, Norway; ^3^Genomics Core Facility, Department of Core Facilities, Institute for Cancer Research, Oslo University HospitalOslo, Norway; ^4^Department of Informatics, University of OsloOslo, Norway; ^5^Institute of Cancer Genetics and Informatics, Oslo University HospitalOslo, Norway; ^6^Institute for Clinical Medicine, Faculty of Medicine, University of OsloOslo, Norway; ^7^Department of Oncology, Oslo University Hospital – The Norwegian Radium HospitalOslo, Norway

**Keywords:** adenocarcinomas, squamous cell carcinomas, large cell carcinomas, *TP53*, never-smoker, mutation spectrum, progression free survival

## Abstract

**Background:** *TP53* mutations are among the most common mutations found in lung cancers, identified as an independent prognostic factor in many types of cancers. The purpose of this study was to investigate the frequency and prognostic impact of *TP53* mutations in never-smokers and in different histological subtypes of lung cancer.

**Methods:** We analyzed tumor tissue from 394 non-small cell carcinomas including adenocarcinomas (*n* = 229), squamous cell carcinomas (*n* = 112), large cell carcinomas (*n* = 30), and others (*n* = 23) for mutations in *TP53* by the use of Sanger sequencing (*n* = 394) and next generation sequencing (*n* = 100).

**Results:** *TP53* mutations were identified in 47.2% of the samples, with the highest frequency (65%) of mutations among squamous cell carcinomas. Among never-smokers, 36% carried a *TP53* mutation, identified as a significant independent negative prognostic factor in this subgroup. For large cell carcinomas, a significantly prolonged progression free survival was found for those carrying a *TP53* mutation. In addition, the frequency of frameshift mutations was doubled in squamous cell carcinomas (20.3%) compared to adenocarcinomas (9.1%).

**Conclusion:** *TP53* mutation patterns differ between the histological subgroups of lung cancers, and are also influenced by smoking history. This indicates that the histological subtypes in lung cancer are genetically different, and that smoking-induced *TP53* mutations may have a different biological impact than *TP53* mutations occurring in never-smokers.

## Introduction

Lung cancer is one of the most common types of cancers, and due to its aggressiveness, this disease is also positioned as the most deadly cancer disease worldwide (Ferlay et al., 2015). Lung cancers can be divided into small-cell lung carcinomas (SCLC) and non-small cell lung carcinomas (NSCLC), consisting of adenocarcinomas (AC), squamous cell carcinomas (SCC), and large cell carcinomas (LCC) (Travis, 2014). The *TP53* gene has been known as a tumor suppressor since the 1990s (Malkin et al., 1990). The *TP53* protein is involved in regulation of essential cell activities, like the cell cycle, cell death, cell differentiation, DNA repair, and formation of blood vessels (Lane and Levine, 2010), and has been called “the guardian of the genome.” These pathways are also involved in processes required to become a cancerous cell, and comprises several of the hallmarks of cancer, such as sustained angiogenesis and evading apoptosis (Hanahan and Weinberg, 2011). Since the first discovery of the protein, much effort has been invested to reveal the spectrum of function for this protein and the related pathways. Still, details about the consequences of the different types of *TP53* mutations for cancer patients are largely unknown. Research has shown that mutations in the *TP53* gene are frequent in almost all types of cancers (Hollstein et al., 1991), and are present in approximately 50% of all NSCLC (Toyooka et al., 2003). Numerous of these mutations may be due to smoking history, and a frequent transversion, GC to TA, is strongly correlated to exposure to carcinogens found in tobacco (Pfeifer et al., 2002).

Many researchers have claimed that mutations in *TP53* are prognostic, or predictive to treatment response, while others have failed to demonstrate this association (Kandioler-Eckersberger et al., 1999; Olivier and Taniere, 2011; Scoccianti et al., 2012). Today, we know that a mutation in the *TP53* gene can affect the protein in many different ways. The missense mutations are the most common type of mutations, leading to production of protein that differs from WT *TP53* by just one amino acid. A growing body of evidence supports the claim that missense mutant *TP53* often have a gain of function (GOF), leading to high expression levels in tumor cells (Goldstein et al., 2011). Deletions and insertions of nucleotides are also common, which often lead to inactive truncated protein. The *TP53* WT can be modified post-translationally in many different ways, such as by methylation, phosphorylation, acetylation, and sumoylation (Nguyen et al., 2014; Rodriguez, 2014), but the effect of such modifications *in vivo* is difficult to assess. Altering the gene *Wrap 53*, an antisense transcript of *TP53*, may also modulate the *TP53* activity (Mahmoudi et al., 2009), depending on type of tissue. Thus, the difficulties in arriving at consistent conclusions may be related to the varied functional consequences of different mutations, leading to heterogeneous p53-related phenotypes.

In order to explore the distribution of *TP53* mutations in lung cancer and their impact on survival in the different histological subgroups, we have investigated *TP53* mutations in 394 non-small cell lung carcinomas, and correlated this with smoking history and clinical data, such as survival, stage, tumor size, *EGFR* mutation status and histology.

## Materials and Methods

The patients in this study were diagnosed with operable NSCLC, and underwent curatively intended surgical resection at Rikshospitalet, Oslo University Hospital, Norway during the period 2006–2011. Clinical data were obtained from questionnaires, medical journals, and histology reports, and follow up information were reported from the patient’s local hospital. The project was approved by the institutional review board and the Regional Ethics Committee (S-06402b). The participants in our study received oral and written information and signed a written consent form before entering the project.

Tumor tissue was dissected from the tumor periphery, containing presumed vital tumor tissue without necrosis. Immediately after dissection, the tumor specimens were snap frozen in liquid nitrogen and stored at -80°C until DNA extraction. EDTA-blood was collected prior to surgery. Totally, 394 tumor specimens consisting of 229 AC, 112 SCC, 30 LCC, and 23 lung cancer carcinomas with other types of histology, such as carcinoids and undifferentiated, were included in this study. All tumor stages were present in the cohort. However, a predominance of stages I and II was included due to inoperable tumors in later stages. Smoking history information revealed that 28 patients (7.1%) were never-smokers, 138 were smokers, and 228 reported to be former-smokers, defined as having quit smoking at least 1 year prior to diagnosis. The never-smokers were mainly diagnosed with AC, only four having a different histology (one SCC, one undifferentiated, two carcinoids).

### DNA Extraction

DNA was extracted from tumor tissue using Maxwell^®^16 DNA Purification Kits and a Maxwell^®^16 instrument. The procedure was performed according to technical manual, Literature # TM284^1^. DNA from blood was isolated using the Master Pure DNA purification Kit for blood according to the DNA Purification Protocol^2^.

### EGFR Mutation Analyses

Tumor specimens were analyzed for *EGFR*-mutations at Unit of Molecular Pathology, Department of Pathology, Oslo University Hospital. The mutation analysis of EGFR exons 18–21 was performed by real-time PCR using TheraScreen EGFR mutation kit (DxS, Manchester, UK), which analyses 28 of the most commonly occurring genetic changes.

### *TP53* Sequencing

The gene *TP53* was analyzed by the Sanger Sequencing method in the 394 tumor samples. The procedure was performed on an Applied Biosystems 3730 DNA analyser according to the supplier’s handbook, Applied Biosystem 3730/3730X/DNA Analysers Part 4331467 Rev.B^3^. All exons from 2 to 11, including the 16 flanking base pairs of each exon, were investigated. Information regarding primers and PCR conditions are displayed in Supplementary Tables 1 and 2. The sequences were aligned and analyzed using SeqScape v.2.5 according to the project template [*TP53* accession nr: NM_000546^4^ (*TP53*ref_NC000017.9_NT010718.15)]. All the sequences were manually and independently evaluated by two persons.

### Mutation Classification

The *TP53* mutations recorded in this study were classified according to their predicted effect on the protein. The different categories were silent, missense in non-DBM, missense in DBM, non-sense, splice, frameshift, and inframe, as previously described (Olivier et al., 2006). For some of the analyses, the categories missense (non-DBM and DBM) and non-missense (frameshift, splice, non-sense, and inframe) were used.

### Next Generation Sequencing (NGS) by Illumina Multiplexed Sequencing

Extracted DNA from 100 tumors (of the 394) and corresponding blood samples from the same patients was sequenced using the Agilent SureSelect Human DNA Kinome panel. This panel includes all known kinases and kinase receptors, supplemented with selected cancer genes. In total, protein-coding and untranslated regions of 612 genes were sequenced. Library construction and in solution capturing was performed following manufacturer’s instructions. Sequencing was performed on an Illumina Genome Analyzer generating paired-end reads of 75 bp in length. Demultiplexing and quality filtering was performed using Illumina’s pipeline. Downstream analysis and detection of somatic variants was performed using an in-house developed pipeline based on GATK (McKenna et al., 2010) and MuTect (Cibulskis et al., 2013). Statistics for *TP53* coverage are included in Supplementary Table 3.

### Statistics

Progression free survival was calculated from the time of surgery until the time of metastasis, local recurrence, death from lung cancer, or death from other reasons (censored). If still alive, the last date of follow up was used (censored). Median PFS values were estimated using Kaplan–Meyer survival analyses and log-rank tests. Univariate and multivariate analyses with Cox regression were used to explore the effect of different factors on PFS. Independent covariates identified as significant (*p* < 0.05) in the univariate model were evaluated in the Cox proportional hazards model. Chi-square test (χ^2^) was used to evaluate the distribution of *TP53* in clinical features (IBM, SPSS, statistics, version 21). A *p*-value <0.05 was considered significant. Mutations spectrum visualization was performed in R version 3.1.3, using gg2plot (R Core Team, 2013).

## Results

### Characterization and Mutation Status of the Study Population

Of the 394 lung cancer patients analyzed for *TP53* mutations, 47.2% (*n* = 186) harbored a *TP53* mutation and 4.5% (*n* = 18) had two different *TP53* mutations. Twenty-nine (7.4%) patients were diagnosed with an *EGFR* mutation, of which 54% were never-smokers and 41% of these also carried a *TP53* mutation in their tumor.

Results from NGS identified six somatic mutations not detected by Sanger sequencing. While two of these mutations resided in the intronic region, not covered by Sanger sequencing in this study, four were presumably missed due to low mutant allele frequency. One deletion in a splice site and one complex exonic mutational event (a deletion combined with either an insertion or a substitution) found by Sanger Sequencing were not reported as somatic by the deep sequencing pipeline. Manual inspection of the deep sequencing data showed that the first case was supported by 4 out of 32 reads, and the second case by 13 out of 48 reads, at their respective locations. In both cases, the variants were misclassified by the variant calling software in spite of their detection by the sequencing technology.

There was no significant difference in the tumor size or stage between the *TP53* mutated and the WT tumors. However, the percentage of *TP53* mutations increased from 44% in stage I to 60% in stage IV, and from 41% in tumor size < 2cm to 53% in tumor size > 7cm. The frequency of *TP53* mutations was significantly differentially distributed across the histological types (*p* < 0.001, χ^2^ test). The SCC and LCC had a higher frequency of *TP53* mutations compared to AC. The tobacco consumption was significantly higher among those carrying a *TP53* mutation (*p* = 0.004, χ^2^ test). More clinical variables associated with *TP53* mutation status are displayed in **Table 1**.

**Table 1:** Association of clinical variables to *TP53* mutation status is listed below.

	Characteristics of the patients	All patients	Patients with *TP53* wt No. (%)	Patients with *TP53* mutation No. (%)	*P*-value ^†^
Sex					0.54
	Male	199	102 (51)	97 (49)	
	Female	195	106 (54)	89 (46)	
Histology					<0.001
	AC never smokers (AC.never)	24	15 (62.5)	9 (37.5)	
	AC	205	121 (59)	84 (41)	
	SCC	112	39 (35)	73 (65)	
	LCC	30	13 (43)	17 (57)	
	Other	23	20 (87)	3 (13)	
Stage					0.55
	IA/IB	222	124 (56)	98 (44)	
	IIA/IIB	109	53 (49)	56 (51)	
	IIIA/IIIB	58	29 (50)	29 (50)	
	IV	5	2 (40)	3 (60)	
Tumor size					0.8
	<2 cm	82	48 (59)	34 (41)	
	2–2.9 cm	124	64 (52)	60 (48)	
	3–4.9 cm	117	62 (53)	55 (47)	
	5–6.9 cm	52	25 (48)	27 (52)	
	>7 cm	19	9 (47)	10 (53)	
Smoking history					0.004
	Never-smoker	28	18 (64)	10 (36)	
	Cigarettes				
	<20 Pack years	75	52 (69)	23 (31)	
	20–39 Pack years	166	85 (51)	81 (49)	
	40–59 Pack years	94	40 (43)	54 (57)	
	>60 Pack years	25	9 (36)	16 (64)	
	Unknown	6	4 (67)	2 (33)	
EGFR status					0.41
	EGFR mutated	29	17 (59)	12 (41)	
	EGFR wild type	345	175 (51)	170 (49)	
	Unknown	20	16 (80)	4 (20)	

*Frequencies are shown both in number (No.) and percent (%). ^†^Pearson’s Chi-square test*.

### New Genetic Variant Identified by NGS

Three patients with the same germline variant in codon 254 (p.I254V), not previously described as a polymorphism, was detected in approximately 50% of the reads in both tumor and blood. All three patients carried an additional somatic mutation in *TP53*. More details are listed in Supplementary Table 4.

### *TP53* Mutation Spectrum in Histological Subtypes and Association with Smoking History

**FIGURE 1:**
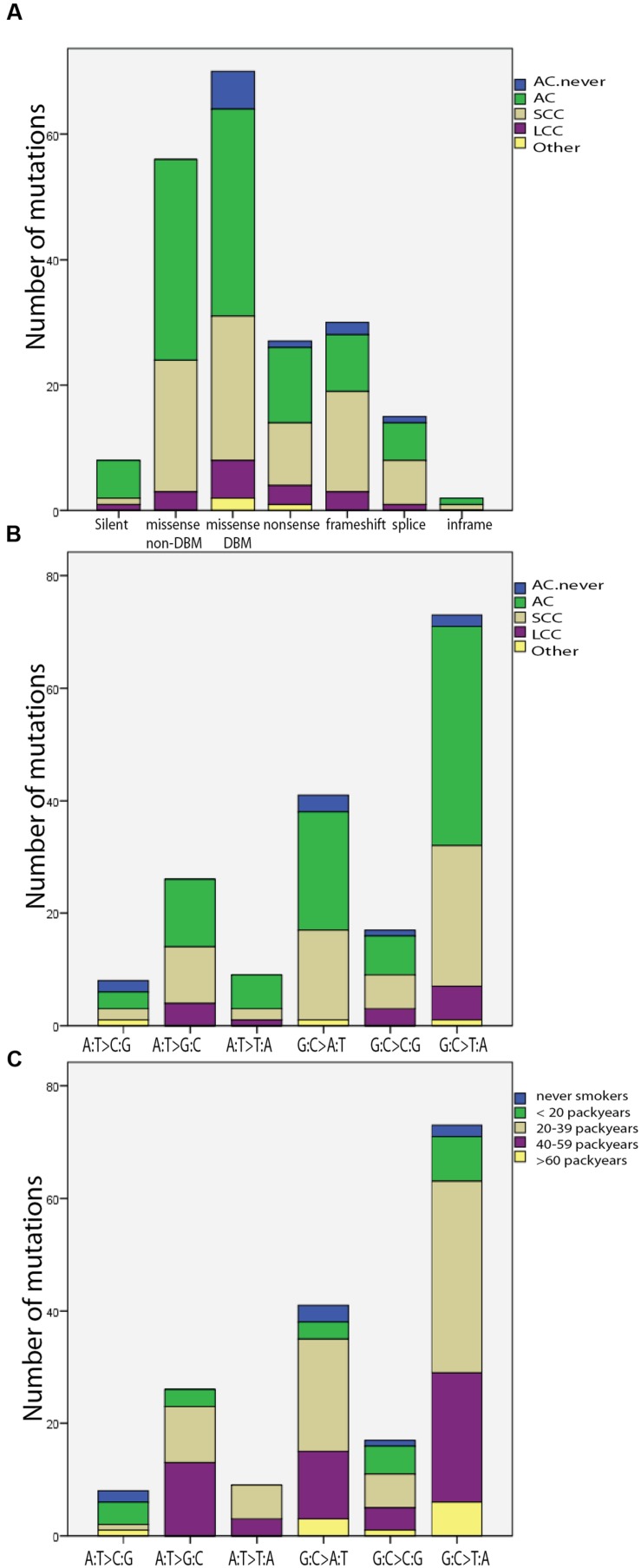
***TP53* mutations in subtypes of lung cancer**. AC with no smoking history are called “AC.never.” Double mutations and silent mutations are included. **(A)** Frequency of mutation type illustrated for the different type of histology. **(B)** Distribution of base change type in the histological subgroups. **(C)** Distribution of base change type in groups with different smoking history.

**FIGURE 2:**
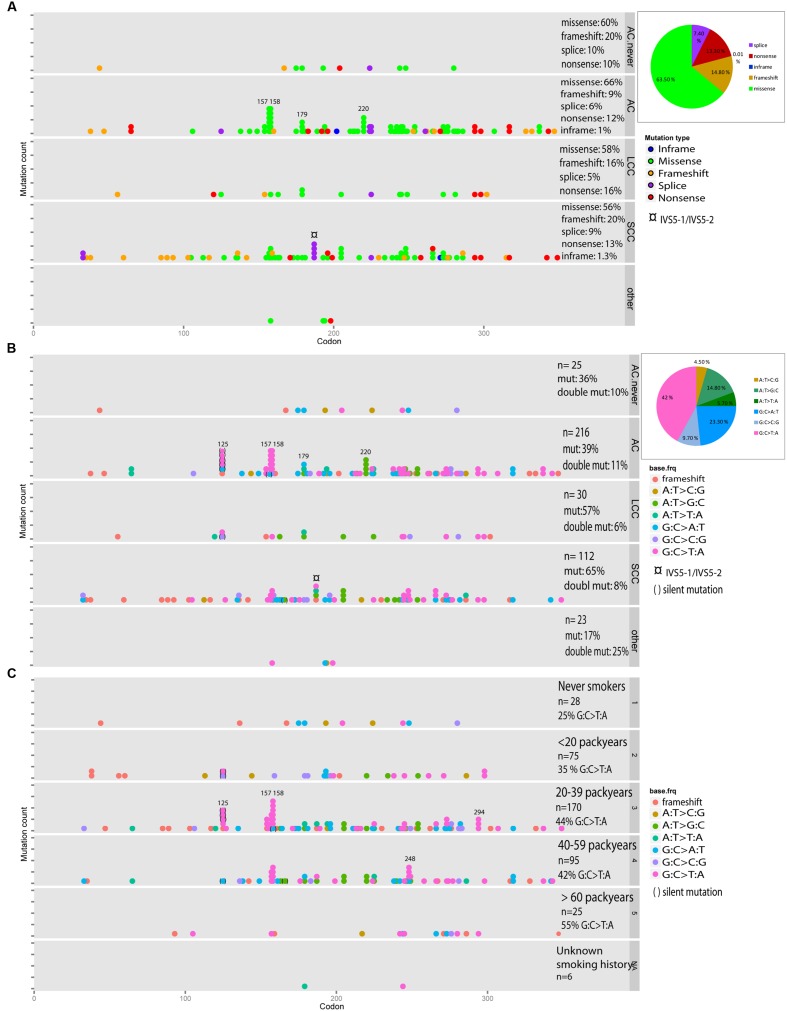
***TP53* mutation spectrum in subtypes of lung cancer**. AC with no smoking history are called “AC.never.” Double mutations are included **(A–C)**, and calculated as the number of double mutations divided by the total number of mutations. Silent mutations are included **(B,C)**. A pie chart shows the fraction of each category. **(A)** Distribution of mutation type across the gene is illustrated for the different types of histology. **(B)** Distribution of base change types in the histological subgroups. **(C)** Distribution of base change types in groups with different smoking history.

In total, 128 (61%) missense mutations, 30 (14.3%) frameshift mutations and 27 (12.9%) non-sense mutations were identified, including double mutations. The 20.3% of the mutations in the SCC samples were frameshift mutations, compared to 9.1% of the mutations in the ACs (*p* = 0.72, χ^2^ test, Supplementary Table 5, **Figures 1A** and **2A**). The most frequently mutated sites were codons 158 (*n* = 11) and 157 (*n* = 8) mainly being G:C > T:A transversions (*n* = 7 and *n* = 9, respectively), and codon 179 (*n* = 7). Among the AC samples, four A:T > G:C transitions were detected at codon 220, and among the SCC samples four splice mutations in intron IVS5-1 and IVS5-2 were discovered (**Figures 1B** and **2B**). The G:C > T:A transversion was the most frequent base change (*n* = 74, 42%), and the incidence significantly increased proportionally with number of pack years (*p* = 0.012, χ^2^ test). This is illustrated in **Figures 1C** and **2C**. Six silent mutations at codon 125 were observed, five of these were AC.

A high frequency of double mutations was observed (*n* = 18, 4.6%, including the germline variant p.I254V). As depicted in **Figure 2B**, the number of double mutations was distributed equally among the different histological subtypes, and 27.8% (*n* = 5) were G:C > T:A transversions. Five of the double mutated samples were also sequenced by next generation sequencing (NGS). The frequencies of the detected mutations are listed in Supplementary Table 6.

In never-smokers, the proportion of G:C > T:A transversion, was 25% (*n* = 2), whereas G:C > A:T transition was most frequently observed (37.5%, *n* = 3). Missense mutations were the most common type of mutation.

### The Impact of *TP53* Mutations on Lung Cancer Survival

**FIGURE 3:**
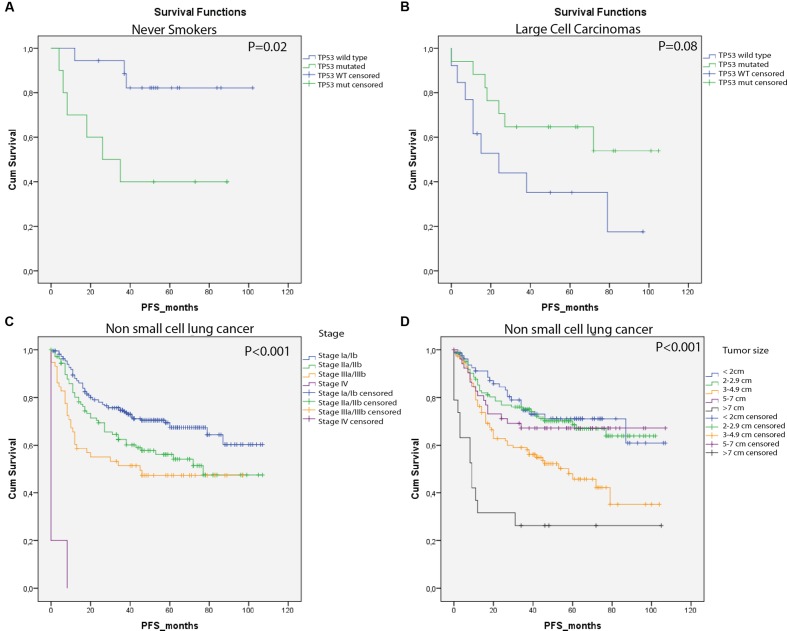
**Kaplan–Meyer estimates of PFS for study patients by group**. **(A)** Never-smokers without *TP53* mutation showed significant prolonged time to progression. **(B)** Individuals with *TP53* mutated large cells carcinomas showed a beneficial PFS compared with the non-carriers. **(C)** Stage was significantly associated with PFS. **(D)** Tumor size was significantly associated with PFS.

In this study, harboring a *TP53* mutation did not affect PFS significantly (HR = 1.24; 95% CI = 0.9–1.72; *p* = 0.19). When stratifying into histological subgroups, patients with LCC seemed to have an increased PFS, when a *TP53* mutation was present (HR = 0.4; 95% CI = 0.2–1.1; *p* = 0.08; **Figure 3B**). After correction for covariates (tumor size and stage) in a multivariate Cox Regression analysis, *TP53* status was an independent predictor of outcome in LCC patients (HR = 0.22; CI = 0.06–0.8, *p* = 0.026, Supplementary Table 7). Patients with *TP53* mutated AC and SCC seemed to have an unfavorable survival, although this was not significant (**Table 2** and Supplementary Table 7). Never-smokers had a significantly reduced PFS when carrying a *TP53* mutation (HR = 5.3, 95% CI = 1.3–21.5, *p* = 0.02, **Figure 3A**). Multivariate Cox Regression analysis confirmed the independent prognostic value of *TP53* mutations for never-smokers (HR = 20.2; 95% CI = 2.8–143.7, *p* = 0.003, Supplementary Table 7). For the different categories of *TP53* mutation, missense mutations in the DBM seem to have the largest impact on PFS (**Table 2**), although not significant. Tumor size and stage showed the strongest prognostic factor (*p* < 0.001, **Figures 3C,D**).

**Table 2:** Univariate Cox regression analysis (SPSS).

	Patient statistics		Univariate Cox regression analysis		
Variables	No. of patients	Events (Censored)	Hazard ratio (HR)	95% CI of HR	*p*-value
Gender	394	148 (246)			
Male	199		1		
Female	195		1.24	0.90–1.72	0.19
*TP53* status	394	148 (246)			
Wild type	208		1		
Mutated	186		1.24	0.90–1.72	0.19
Histology	394	148 (246)			
AC	229		1		
SCC	112		0.99	0.69–1.44	0.97
LCC	30		1.4	0.83–2.41	0.21
other	23		0.29	0.09–0.93	0.037
*TP53* status; AC	229	88 (141)			
Wild type	136		1		
Mutated	93		1.29	0.85–1.96	0.24
*TP53* status; SCC	112	41 (71)			
Wild type	39		1		
Mutated	73		1.32	0.68–2.56	0.41
*TP53* status; LCC	30	16 (14)			
Wild type	13		1		
Mutated	17		0.44	0.15–1.12	0.08
*TP53* status; other	23	3 (19)			
Wild type	20		1		
Mutated	3		3.42	0.31–37.91	0.32
*TP53* status; never smokers	28	9 (19)			
Wild type	18		1		
Mutated	10		5.3	1.32–21.5	0.02
Stage	394	148 (246)			
I	222		1		
II	109		0.025	0.01–0.07	<0.001
III	58		0.04	0.02–0.11	<0.001
IV	5		0.055	0.02–0.15	<0.001
Tumor size	394	148 (246)			
<2 cm	82		1		
2–2.9 cm	124		0.19	0.10–0.37	<0.001
3–4.9 cm	116		0.21	0.11–0.39	<0.001
5–6.9 cm	53		0.39	0.22–0.72	<0.002
>7 cm	19		0.23	0.12–0.48	<0.001
*EGFR* status	374	143 (231)			
Wild type	345		1		
Mutated	29		0.78	0.41–1.49	0.46
Type of mutations	394	148 (246)			
WT/silent	208		1		
Missense non-DBM	48		0.89	0.57–1.38	0.6
Missense DBM	67		0.9	0.49–1.66	0.74
Non-sense/frameshift/splice	71		1.39	0.83–2.32	0.21

## Discussion

In the present study, 47.2% of the lung cancers harbored a *TP53* mutation. With respect to the different types of histology, the mutation frequency was highest among patients with SCC, while those with AC and others showed the lowest frequency. In the LUAD study, 46% of the AC harbored a *TP53* mutation and 81% were recorded with a smoking history (Cancer Genome Atlas Research Network, 2014), which is in line with present results. However, even if a high frequency of *TP53* mutations was observed among SCC in our study, it’s less than what was reported in the LUSQ study (Cancer Genome Atlas Research Network, 2012), where 81% of 178 SCCs harbored a *TP53* mutation. In present study, 84% of the samples were recorded as stage I/II, and a tendency of higher mutation rate in higher stages was seen. The discrepancy between the two studies might be explained by few late stage samples analyzed in present cohort. In addition, in another study of 110 SCC a frequency of *TP53* mutations on 57% was found (Scoccianti et al., 2012), and in a meta-study of SCLC and NSCLC, the frequency of *TP53* mutations was 40% among smokers (*n* = 1232), which is more in line with our findings (Liu et al., 2014). In present study, a lower frequency of *TP53* mutations was seen among patients with *EGFR* mutations (41%), and among never-smokers (36%), mainly diagnosed with AC.

The frequency of *TP53* mutations increased with tobacco consumption. For the never-smokers, 36% of the patients were *TP53* mutated compared to 64% among those reported with more than 60 pack-years. A study of somatic mutations in AC has previously reported that smokers harbor a higher number of mutations in genes, such as KRAS and STK11, compared to never-smokers (Ding et al., 2008). *TP53* mutations are common in lung cancer, and occur more frequently in smokers than in never-smokers (Sun et al., 2007). In addition, never-smokers with *TP53* WT showed beneficial PFS. This was not seen among the smokers, who may have a lot of other mutations with impact on the survival. A higher accumulation of mutations in genes like *NTRK2*, *EPHA7*, *PPKCG*, and *FLT4* in AC with higher stage has been reported in a study of lung AC (Ding et al., 2008). The percentage of *TP53* mutations increased from 44% in stage I to 60% in stage IV. If a higher number of stage IV samples were present in our study, this tendency might have reached significance. A tendency toward more *TP53* mutations in the larger tumors was also seen.

### Identification of a New Variant in *TP53*

Germline mutations in *TP53* are closely linked to the condition called Li–Fraumeni syndrome (LFS), with an early onset of tumorigenesis. Three patients in our cohort revealed a missense alteration in exon 7 (p.I254V), both in tumor tissue and in blood. According to Bougeard et al. (2008), mutation in codon 254 (p.I254L) has been reported inherited in one LFS family in France, and p.I254V has been reported in six times as somatic mutation, but is not a known polymorphism (International Agency for Research on Cancer, 2015). Nevertheless, this variant has been recorded in the ExAC database as a germline variant in 1 out of 121322 alleles with a frequency of 8.24*E* – 06 (Lek et al., 2015). Unfortunately, phenotype information is not available and information regarding cancer status is not known. In a functional analysis in yeast, the predicted protein change p.I254V was not shown to be functionally defective (Kato et al., 2003; International Agency for Research on Cancer, 2015). In our study, the three patients did not have an early tumor onset, or a family history of cancer. Thus, this sequence variant might be an un-described polymorphism, with no influence on tumorigenesis. The mutation was present in approximately 50% of the reads in NGS, which further supports that it is a polymorphism. However, since each of the three patients harbors an additional somatic mutation in the *TP53* gene, and the p.I254V variant was not found among those with *TP53* WT, we might speculate that this germline missense variant may affect the susceptibility of acquiring somatic mutations in the same gene. If so, this might also explain why this germline variant was found in a much higher frequency in our cohort compared to the ExAC database. In addition, the ExAC database may contain a higher frequency of certain disease associated variants (Lek et al., 2015).

### The *TP53* Mutation Spectrum Has Different Implications in Different Subgroups

*TP53* mutation categories were un-evenly distributed in the histological subgroups. The categories missense, frameshift, splice, and non-sense were present in all histological subtypes. Interestingly, a substantially higher frequency of frameshift mutations was seen in SCC compared to AC. This did not reach significance, which may be due to only 30 cases reported with frameshift in total. Missense mutations (DBM and non-DBM combined) were more frequent within the AC-group than among SCCs and LCCs. This may indicate that *TP53* mutations in lung cancer can have different impact for the different types of histology, also reflected in the survival analysis. Interestingly, the survival was significantly better for patients with LCC harboring *TP53* mutations. For patients with AC and SCC, an opposite tendency was found. It is well known that the groups of histology often show different clinical features, and also the *TP53* mutations may have different implications for the different histological subtypes. In a large study of breast cancer patients, *TP53* mutations have been identified as an independent factor of poor prognosis (Olivier et al., 2006). This may not be the case for lung cancer patients since they generally harbor a large number of mutations due to smoking and carcinogen exposure. Among the never-smokers who usually are recognized with fewer mutations, a *TP53* mutation was an independent prognostic factor of poor PFS. However, the multivariate analysis contained few individuals and must be interpreted with caution.

In this study, patients with missense mutations in the DNA binding motif seemed to have the poorest prognosis. Poeta et al. (2007) found that disruptive *TP53* mutations were associated with significantly decreased overall survival in head and neck cancers. This is in contrast to a study performed on advanced NSCLC, where having non-disruptive *TP53* mutations was an independent prognostic factor of reduced survival (Molina-Vila et al., 2014). The SCC and LCC revealed the highest frequency of *TP53* mutations, and the number of mutations increased with tobacco consumption. The transversion G:C > T:A, is a frequent base change in smokers, representing 42% of the base changes in this study. This transversion occurred equally in the different types of histology, and did not impact survival. However, among never-smokers the frequency was only 25%, and the G:C > A:T transition was the most frequent base change, consistent with other studies (Pfeifer et al., 2002; Liu et al., 2014). Interestingly, the number of G:C > T:A transversions increased with the number of pack years, especially after 20 pack years. We also observed that G:C > T:A transversions tended to cluster in hotspots, such as codons 157, 158, and 248, although not in never-smokers. Codons 157, 158, and 248 have been registered as smoking associated hotspot regions in lung cancer in previous studies (Pfeifer et al., 2002; Gibbons et al., 2014). However, the hotspots for AC were slightly different from the hotspot in SCC. Four missense mutations in codon 220 were observed only in AC, while an increased frequency of splice variants in IVS5-1/IVS5-2 was seen only in SCC.

## Conclusion

The *TP53* mutation profiles were different in the specific histologies and smoking categories, with a high number of frameshift variants in SCC. LCC with *TP53* WT showed a significantly reduced survival, with an opposite tendency for ACC and SCC. The *TP53*-mutations in never-smokers differed from that of ever smokers, supporting the claim that lung cancer in never-smokers is a separate entity in lung cancer.

## Author Contributions

AH, ÅH, A-LB-D, and OB designed the study. ÅH and OB are responsible for clinical input. AH, PV, and CS performed the Sanger sequencing and interpretation of the data. AH and LS-P did the statistical analysis. LM-Z, DV, EH, and OM conducted the NGS data analysis and interpretation of the NGS data. AH and ÅH drafted the manuscript. All authors read, revised the manuscript critically and approved the final manuscript.

## Conflict of Interest Statement

The authors declare that the research was conducted in the absence of any commercial or financial relationships that could be construed as a potential conflict of interest. The reviewer HD and handling Editor declared their shared affiliation, and the handling Editor states that the process nevertheless met the standards of a fair and objective review.

## Footnotes

1 http://www.promega.com.

2 http://www.epicentre.com.

3 http://www.appliedbiosystems.com.

4 http://www.ncbi.nlm.nih.gov/nuccore/NM_000546

## ABBREVIATIONS

AC: adenocarcinomas
DBM: DNA binding motif
LCC: large cell carcinomas; non-DBM
LCC: large cell carcinomas
non-DBM: non-DNA binding motif
NSCLC: non-small cell lung cancers
PFS: progression free survival
SCC: squamous cell carcinomas
SCLC: small cell lung cancers
WT: wild-type
