# Supplementary material for: TP53 Mutation Spectrum in Smokers and Never Smoking Lung Cancer Patients
Source: Front Genet. 2016 May 11;7:85. doi: 10.3389/fgene.2016.00085 (PMC4863128; doi:10.3389/fgene.2016.00085)
Supplement: Supplementary file 1 [file Supplementary_Tables.DOCX]

**SUPPLEMENTARY**

Table 1 Primer sequences for PCR amplification and cycle sequencing

| **Primer sequences for PCR amplification** | | | |
| --- | --- | --- | --- |
| **Exons** | **Forward primer (5’)** | **Reverse primer (3’)** | **PCR fragment length (Bp)** |
| 2,3 | GGAGTGCTTGGGTTGTGGT | CGGCAAGGGGGACTGTA | 586 |
| 4 | GACTTCCTGAAAACAACG | CACACATTAAGTGGGTAAAC | 593 |
| 5,6 | TTT CTT TGC TGC CGT CTTC | TTG CAC ATC TCA TGG GGT TA | 588 |
| 7 | GAC CAT CCT GGC TAA CGG | CAC AGG TTA AGA GGT CCC AAA | 595 |
| 8,9 | TTT GGG ACC TCT TAA CCT GT | CAG GCA AAG TCA TAG AAC CAT | 733 |
| 10 | CAT GTT GCT TTT GTA CCG TC | GGC AAG AAT GTG GTT ATA GGA | 396 |
| 11 | AAG GGA AGA TTA CGA GACT | TAA GCT GGT ATG TCC TAC TC | 500 |
| **Primer sequences for cycle sequencing** | | | |
| o    M13 forward primer sequence: 5'-TGTAAAACGACGGCCAGT-3' | | |  |
| o    M13 reverse primer sequence: 5'-CAGGAAACAGCTATGACC-3' | | |  |

Table 2 Time and temperature conditions during A: PCR amplification, and B: cycle sequencing

| **Stage** | **A. 96 Thermal Cycler (Applied Biosystems)** | | **Stage** | **B. 96 Thermal Cycler (Applied Biosystems)** | |
| --- | --- | --- | --- | --- | --- |
|  | **Temp** | **Time** |  | **Temp** | **Time** |
| Hold | 96°C | 5 min | Hold | 37°C | 15 min |
| Cycle | 94°C | 30 sec | Hold | 80°C | 2 min |
| (35 cycles) | 62°C* | 45 sec | Hold | 96°C | 1 min |
|  | 68°C | 45 sec | Cycle | 96°C | 10 sec |
| Hold | 72°C | 2 min | (25 cycles) | 50°C | 5 sec |
| Hold | 4°C | ∞ |  | 60°C | 4 min |
|  |  |  | Hold | 4°C | ∞ |

Table 3 HTC sequencing statistics for *TP53.* Target Coverage >=30_Percent is percentage (%) of bases within the targeted kinome regions, which had non-duplicate read coverage of at least 30% and Target Coverage >=100_Percent is the percentage (%) of bases within the targeted kinome regions, which had non-duplicate read coverage of at least 100%. Data is shown for both tumour and normal samples.

| **Tumor samples** | | |  |  |
| --- | --- | --- | --- | --- |
| Statistic | | Mean coverage | Target Coverage >=30 (%) | Target Coverage >=100 (%) |
| Minimum | | 14.88 | 4.9 | 0 |
| 1st_Quartile | | 37.37 | 55.9 | 0 |
| Median | | 50.21 | 83.3 | 4.9 |
| Mean | | 86.82 | 73.68 | 22.03 |
| 3rd_Quartile | | 72.19 | 97.2 | 20.4 |
| Maximum | | 619.37 | 100 | 100 |
|  | |  |  |  |
| **Normal samples** | | |  |  |
| Statistic | Mean  coverage | | Target Coverage >=30 (%) | Target Coverage >=100 (%) |
| Minimum | 22.42 | | 24.9 | 0 |
| 1st_Quartile | 31.69 | | 48.6 | 0 |
| Median | 39.04 | | 62.6 | 0 |
| Mean | 87.77 | | 69.05 | 21.14 |
| 3rd_Quartile | 69.08 | | 97.1 | 21.4 |
| Maximum | 492.88 | | 100 | 100 |

Table 4 Clinical data of the patients with detected *TP53* germline variants. The blood sample and tumor sample from the female patient were analyzed with Sanger Sequencing. All the other samples were analyzed with both techniques (NGS and Sanger Sequencing).

| ***Sex*** | ***Male*** | ***Male*** | ***Female*** |
| --- | --- | --- | --- |
| ***TP53 mutation, germline (blood)*** | *c.254 ATC>GTC (p.I254V)* | *c.254 ATC>GTC (p.I254V)* | *c.254 ATC>GTC (p.I254V)* |
| ***Number of reads tumor*** | *36 of 66 (0.55)* | *24 of51 (0.47)* | *No NGS data* |
| ***TP53 mutation, somatic (tumour tissue)*** | *c.181 CGC>CAC (p.R181H)* | *c.158 CGC>CTC (p.R158L)* | *1 b.p deletion c.38 (frameshift)* |
| ***Number of reads tumor*** | *18 of 59* | *11 of 46* | *No NGS data* |
| ***Histology*** | *Adenocarcinoma* | *Adenocarcinoma* | *Squamous Cell Carcinoma* |
| ***Tumour size*** | *1.9cm* | *4.5cm* | *2.4cm* |
| ***Stage*** | *I* | *I* | *III* |
| ***Survival months after surgery*** | *Still alive (4 years)* | *Dead after 21 months* | *Still alive (2 years)* |
| ***Smoking History*** | *Current smoker, 35 pack years* | *Former smoker, 27 pack years* | *Former smoker, 9 pack years* |

Table 5 A: The different types of base changes show that the transversion G:C>T:A is equally distributed among the types of histology. The lowest frequency of G:C>T:A is among AC.never. B: Base changes are significantly differentially distributed across smoking history (p=0.012). The distribution of the transversion G:C>T:A is 25% among never-smokers and doubled among those recorded with more than 60 packyears. C: Type of mutation is not differentially distributed across type of histology. Frameshift mutations seem to be unevenly distributed between AC and SCC.

| 1. Pearson Chi-square p=0.13 | | | Base change frequency across type of histology | | | | | Total |
| --- | --- | --- | --- | --- | --- | --- | --- | --- |
|  |  |  | AC.never | AC | SCC | LC | other |  |
|  | A:T>C:G | Number | 2 | 3 | 2 | 0 | 1 | 8 |
|  |  | Percent | 25.0% | 3.4% | 3.3% | 0.0% | 33.3% | 4.5% |
|  | A:T>G:C | Number | 0 | 12 | 10 | 4 | 0 | 26 |
|  |  | Percent | 0.0% | 13.6% | 16.4% | 25.0% | 0.0% | 14.8% |
|  | A:T>T:A | Number | 0 | 6 | 2 | 2 | 0 | 10 |
|  |  | Percent | 0.0% | 6.8% | 3.3% | 12.5% | 0.0% | 5.7% |
|  | G:C>A:T | Number | 3 | 21 | 16 | 0 | 1 | 41 |
|  |  | Percent | 37.5% | 23.9% | 26.2% | 0.0% | 33.3% | 23.3% |
|  | G:C>C:G | Number | 1 | 7 | 6 | 3 | 0 | 17 |
|  |  | Percent | 12.5% | 8.0% | 9.8% | 18.8% | 0.0% | 9.7% |
|  | G:C>T:A | Number | 2 | 39 | 25 | 7 | 1 | 74 |
|  |  | Percent | 25.0% | 44.3% | 41.0% | 43.8% | 33.3% | 42.0% |
| Total | | Number | 8 | 88 | 61 | 16 | 3 | 176 |
|  |  | Percent | 100.0% | 100.0% | 100.0% | 100.0% | 100.0% | 100.0% |
| 1. Pearson Chi-square p=0.012 | | | Base change frequency across smoking history | | | | | Total |
|  |  |  | never | <20 | 20-39 | 40-59 | ≥60 |  |
|  | A:T>C:G | Number | 2 | 4 | 1 | 0 | 1 | 8 |
|  |  | Percent | 25.0% | 17.4% | 1.3% | 0.0% | 9.1% | 4.6% |
|  | A:T>G:C | Number | 0 | 3 | 10 | 13 | 0 | 26 |
|  |  | Percent | 0.0% | 13.0% | 13.0% | 23.6% | 0.0% | 14.9% |
|  | A:T>T:A | Number | 0 | 0 | 6 | 3 | 0 | 9 |
|  |  | Percent | 0.0% | 0.0% | 7.8% | 5.5% | 0.0% | 5.2% |
|  | G:C>A:T | Number | 3 | 3 | 20 | 12 | 3 | 41 |
|  |  | Percent | 37.5% | 13.0% | 26.0% | 21.8% | 27.3% | 23.6% |
|  | G:C>C:G | Number | 1 | 5 | 6 | 4 | 1 | 17 |
|  |  | Percent | 12.5% | 21.7% | 7.8% | 7.3% | 9.1% | 9.8% |
|  | G:C>T:A | Number | 2 | 8 | 34 | 23 | 6 | 73 |
|  |  | Percent | 25.0% | 34.8% | 44.2% | 41.8% | 54.5% | 42.0% |
| Total | | Number | 8 | 23 | 77 | 55 | 11 | 174 |
|  |  | Percent | 100.0% | 100.0% | 100.0% | 100.0% | 100.0% | 100.0% |
| 1. Pearson Chi-square p=0.718 | | | Type of *TP53* mutation distributed across histology | | | | | Total |
|  |  |  | AC.never | AC | SCC | LCC | other |  |
|  | Silent mutation | Number | 0 | 6 | 1 | 1 | 0 | 8 |
|  |  | Percent | 0.0% | 6.1% | 1.3% | 5.3% | 0.0% | 3.8% |
|  | missense non-DBM | Number | 0 | 32 | 21 | 3 | 0 | 56 |
|  |  | Percent | 0.0% | 32.3% | 26.6% | 15.8% | 0.0% | 26.7% |
|  | missense DBM | Number | 6 | 33 | 23 | 8 | 2 | 72 |
|  |  | Percent | 60.0% | 33.3% | 29.1% | 42.1% | 66.7% | 34.3% |
|  | nonsense | Number | 1 | 12 | 10 | 3 | 1 | 27 |
|  |  | Percent | 10.0% | 12.1% | 12.7% | 15.8% | 33.3% | 12.9% |
|  | frameshift | Number | 2 | 9 | 16 | 3 | 0 | 30 |
|  |  | Percent | 20.0% | 9.1% | 20.3% | 15.8% | 0.0% | 14.3% |
|  | splice | Number | 1 | 6 | 7 | 1 | 0 | 15 |
|  |  | Percent | 10.0% | 6.1% | 8.9% | 5.3% | 0.0% | 7.1% |
|  | inframe | Number | 0 | 1 | 1 | 0 | 0 | 2 |
|  |  | Percent | 0.0% | 1.0% | 1.3% | 0.0% | 0.0% | 1.0% |
| Total |  | Number | 10 | 99 | 79 | 19 | 3 | 210 |
|  |  | Percent | 100.0% | 100.0% | 100.0% | 100.0% | 100.0% | 100.0% |

Table 6 Five of the double mutated samples were analyzed by NGS. Type of mutation, codon change, protein change, prediction of the mutation and frequency are displayed in the table.

| **Sample** | **codon change** | **protein change** | **Predicted** | **Frequency reads (%)** |
| --- | --- | --- | --- | --- |
| *DBL.1* | *GAG>TAG* | *p.E271X* | *nonsense* | *9 of 35(0.26)* |
| *DBL.1* | *CAG>TAG* | *p.Q192X* | *nonsense* | *13 of 72 (0.18)* |
| *DBL.2* | *CGG>CAG* | *p.R248Q* | *Missense DBM* | *9 of 31 (0.29)* |
| *DBL.2* | *CAT>TAT* | *p.H179Y* | *Missense DBM* | *6 of 23 (0.26)* |
| *DBL.3* | *CGC>CAC* | *p.R181H* | *Missense DBM* | *18 of 59 (0.31)* |
| *DBL.3* | *ATC>GTC* | *p.I254V* | *missense non-DBM* | *36 of 66 (0.55)* |
| *DBL.4* | *CGC>CTC* | *p.R158L* | *missense non-DBM* | *11 of 46 (0.24)* |
| *DBL.4* | *ATC>GTC* | *p.I254V* | *missense non-DBM* | *24 of 51 (0.47)* |
| *DBL.5* | *TCC>TCT* | *p.S149Y* | *missense non-DBM* | *10 of 20 (0.5)* |
| *DBL.5* | *CGC>CCC* | *p.R158P* | *missense non -DBM* | *10 of 18 (0.56)* |

Table 7 Multivariate Cox regression analysis was performed on the different types of histology and for the never-smokers.

| Multivariate Cox Regression analysis | | | | |
| --- | --- | --- | --- | --- |
| **Never-smokers**  **n=28, events=9** | **Variable** | **Hazard Ratio** | **95% Ci** | **p-value** |
| *TP53* mutation | Wild type | 1 |  |  |
|  | Mutated | 67.8 | 3.95-1164 | 0.004 |
| Tumour size | < 2cm | 1 |  | 0.15 |
|  | 2-2.9cm | 1.86 | 0.17-19.9 | 0.61 |
|  | 3-4.9cm | 28.2 | 1.23-646.8 | 0.04 |
|  | 5-6.9cm | 42.9 | 1.03-1789 | 0.05 |
|  | >7cm |  |  |  |
| Stage | I | 1 |  | 0.6 |
|  | II | 0.48 | 0.05-4.86 | 0.54 |
|  | III | 0.42 | 0.06-2.89 | 0.38 |
|  | IV |  |  |  |
| **Adenocarcinomas**  **n=229, events= 88** | **Variable** | **Hazard Ratio** | **95% Ci** | **p-value** |
| *TP53* mutation | Wild type | 1 |  |  |
|  | Mutated | 1.34 | 0.88-2.05 | 0.171 |
| Tumour size | < 2cm | 1 |  | 0.009 |
|  | 2-2.9cm | 1.38 | 0.73-2.63 | 0.327 |
|  | 3-4.9cm | 2.19 | 1.17-4.12 | 0.01 |
|  | 5-6.9cm | 0.8 | 0.29-2.22 | 0.66 |
|  | >7cm | 3.66 | 1.34-9.98 | 0.01 |
| Stage | I | 1 |  | <0.001 |
|  | II | 1.98 | 1.15-3.41 | 0.013 |
|  | III | 1.78 | 1.01-3.13 | 0.05 |
|  | IV | 311.95 | 31.9-3044 | <0.001 |
| **Sqamous cell carcinomas n=112, events= 41** | **Variable** | **Hazard Ratio** | **95% Ci** | **p-value** |
| *TP53* mutation | Wild type | 1 |  |  |
|  | Mutated | 1.7 | 0.82-3.53 | 0.16 |
| Tumour size | < 2cm | 1 |  | 0.014 |
|  | 2-2.9cm | 0.46 | 0.16-1.36 | 0.16 |
|  | 3-4.9cm | 1.11 | 0.45-2.71 | 0.83 |
|  | 5-6.9cm | 0.4 | 0.01-1.64 | 0.21 |
|  | >7cm | 3.12 | 0.76-12.8 | 0.12 |
| Stage | I | 1 |  | 0.06 |
|  | II | 1.03 | 0.39-2.71 | 0.95 |
|  | III | 1.89 | 0.71-5.06 | 0.2 |
|  | IV | 15.87 | 1.42-178 | 0.03 |
| **Large cell carcinomas**  **n=30, events= 16** | **Variable** | **Hazard Ratio** | **95% Ci** | **p-value** |
| *TP53* mutation | Wild type | 1 |  |  |
|  | Mutated | 0.22 | 0.06-0.83 | 0.03 |
| Tumour size | < 2cm | 1 |  | 0.28 |
|  | 2-2.9cm | 0.81 | 0.05-14.3 | 0.88 |
|  | 3-4.9cm | 2.89 | 0.29-29.1 | 0.37 |
|  | 5-6.9cm | 12.9 | 1.0-166 | 0.05 |
|  | >7cm | 11.5 | 0.72-183 | 0.08 |
| Stage | I | 1 |  | 0.11 |
|  | II | 0.57 | 0.06-5.29 | 0.62 |
|  | III | 0.71 | 0.11-4.5 | 0.72 |
|  | IV | 24.7 | 0.52-1172 | 0.1 |
